# Loxoprofen Sodium Versus Diclofenac Potassium for Post-Dental Extraction Pain Relief: A Randomized, Triple-Blind, Clinical Trial

**DOI:** 10.3390/dj8010002

**Published:** 2019-12-25

**Authors:** Ibrahim Nourwali, Arwa Namnakani, Majd Almutairi, Anas Alaufi, Yasser Aljohani, Saba Kassim

**Affiliations:** 1 Department of Oral and Maxillofacial Surgery, Taibah University Dental College & Hospital, Naif Ibn Abdulaziz Road, Al-Madinah Al-Munawwarah 42353, Saudi Arabia; 2 Interns, Taibah University Dental College & Hospital, Naif Ibn Abdulaziz Road, Al-Madinah Al-Munawwarah 42353, Saudi Arabia; d.arwa.nmk@taibahu.edu.sa (A.N.); jod.taibahu.sa@taibahu.edu.sa (M.A.); anasalaufi@taibahu.edu.sa (A.A.); yaaseer-11@taibahu.edu.sa (Y.A.); 3 Department of Preventive Dental Sciences, Taibah University Dental College & Hospital, Prince, Naif Ibn Abdulaziz Road, Al-Madinah Al-Munawwarah 42353, Saudi Arabia; saba262003@gmail.com or

**Keywords:** analgesics, pain, efficacy, oral, tooth extraction

## Abstract

One of the most common post-operative complications of tooth extraction is pain. Oral analgesics, namely loxoprofen sodium and diclofenac potassium, are often prescribed; however, the efficacy of these drugs irrespective of gender and type of extraction has not been tested. Therefore, this study aimed to compare the efficacy of these two drugs in post-dental extraction pain relief among male and female patients in cases of simple and surgical tooth extraction. A single-center, triple-blind, randomized clinical trial was conducted among 100 male and female patients who underwent tooth extraction at Taibah University Dental College and Hospital in Al-Madinah, Saudi Arabia. The patients reported their pain post-operatively after 6 hours and every 12 h for 3 days using the Verbal Descriptor Scale (e.g., “no pain”, “mild pain”). Descriptive statistics and chi-square tests were run to analyze the data. An equal number of patients received either the drug loxoprofen sodium or diclofenac potassium and completed the study follow-up. Patients allocated to the diclofenac potassium drug group after 36 h were statistically significantly in their reporting of “no pain” and “mild pain” compared to patients allocated to the loxoprofen sodium group (86% vs. 66%, respectively; *p* = 0.019), irrespective of gender or type of tooth extraction. However, both groups demonstrated comparable (*p* > 0.05) post-operative pain relief over the other aforementioned allocated time intervals. In conclusion, the diclofenac potassium group had slightly better control over post-operative pain than the group receiving loxoprofen sodium.

## 1. Introduction

Tooth extraction remains a common procedure in dentistry. The most common cause for tooth extraction is dental caries [1], though teeth are extracted for many other reasons, including severe periodontal diseases, orthodontic correction, unrestorable teeth and supernumerary and impacted teeth. In addition, questionable teeth are often extracted in patients who are going to receive radiation therapy in cases of oral, head or neck cancer. Thus, extraction of abnormal or diseased teeth may lead to an improvement in patient health [2]. Tooth extraction has been linked to oral hygiene, level of education, socioeconomic status and individual lifestyle [1]. However, a common post-operative complication of extraction is tissue damage that results in severe pain. In clinical practice, acute post-operative pain caused by inflammation or tissue damage could prevent patients from completing their treatments in dental clinics [3]. Therefore, it is important to control post-operative pain and inflammation after tooth extraction to improve patient satisfaction. It also plays an important role in cost effectiveness and use of healthcare resources [4].

A recent study indicated that the intensity of pain reaches its maximum score in the first 24 h after surgical tooth extraction using the McGill Pain Questionnaire [5]. The highest pain point occurs between 6 and 8 h after extraction with conventional local anesthetic techniques, as a result of trauma to the hard and soft tissues caused by surgical dental extraction. Post-operative pain intensity can be decreased with the use of a pre-operative analgesic, as the amount of pain triggers prostaglandins discharged into the site of the injuries is reduced. Accumulation of prostaglandins released from the injured tissues increases over time and leads to the amplification of pain intensity [6]. Prostaglandins play a vital role in mediating inflammatory pain by lowering the pain threshold of polymodal transient vanilloid receptors (TRPV1) [7]. TRPV1 receptors are stimulated by various high-intensity stimuli (mechanical, chemical, temperature). TRPV1 are located in dental pulp and on blood vessels supplying the gums [8]. Therefore, the class of nonsteroidal anti-inflammatory drugs (NSAIDs), such as ibuprofen, are the most commonly prescribed analgesics for mild post-operative pain. Ibuprofen showed better dental pain relief compared to paracetamol and placebos [7]. However, it should be noted that for immediate pain relief, doctors may change the medication dosage based on patient health status [9].

Lack of dental pain management guidelines, lack of awareness among dentists and a lack of prescription controls for pain medications in practice cause dentists to fall victim to pharmaceutical companies, and “trial-and-error” became the predominant practice. This subjective method of prescribing medication needs to be replaced by safe prescribing practices that take into account patient variability and include a discussion about the type of dental procedure and effective pain relief to provide the best care for patients [10].

Notably, in Saudi Arabia, diclofenac potassium was found to be more effective than paracetamol or ibuprofen for moderate to severe post-operative pain [6]. The limitation of the aforementioned study was the sample size and that it included only male patients, which may have affected the study results [6]. It has been reported that females have a higher pain rating, less tolerance of noxious stimuli than males and a greater ability to discriminate pain. This means that females have lower thresholds of pain than males. For endogenous pain, or pain of an unknown cause, males generally report fewer multiple pains in more body regions than females [11].

Two oral analgesics were chosen in this study to elucidate the most effective analgesics prescribed by dentists for reducing pain after tooth extraction; they were also chosen for their efficiency, potency and availability in pharmacies for all patients in Saudi Arabia, specifically Al-Madinah. The two randomly distributed analgesics used in this study were loxoprofen (Roxonin 60 mg) and diclofenac potassium (Rapidus 50 mg). Loxoprofen 60 mg is a tablet that contains loxoprofen sodium hydrate as an active ingredient. It is intended for relief of inflammation and pain in rheumatoid arthritis, osteoarthritis, toothache and acute upper respiratory tract inflammation as well as relief of post-operative, post-traumatic or post-dental extraction pain and inflammation [12]. Diclofenac potassium 50 mg is a tablet that contains diclofenac potassium as an active ingredient. Diclofenac potassium tablets are generally used to relieve pain and swelling (inflammation) in various mild to moderate painful conditions such as dental pain, back pain, general pain and swelling, gout attacks, sports injuries and muscle aches [6].

The aim of this study was to assess the efficacy of orally administered loxoprofen sodium compared with diclofenac potassium, among male and female patients with simple and surgical tooth extraction, on post-dental extraction pain. The hypothesis in this study was that no difference would be found between diclofenac potassium and loxoprofen sodium oral analgesics for controlling post-dental extraction pain irrespective of gender and type of extraxtion (simple or surgical).

## 2. Materials and Methods

### 2.1. Study Design, Setting and Sample Size Calculation

This was a single-center, triple-blinded, randomized clinical trial in two groups that took place at Taibah University Dental College and Hospital in Al-Madinah, Saudi Arabia, over the period between February 2018 and March 2018. Based on previous studies and the formula provided on the OpenEpi website, the sample should include 50 participants. In this study, the sample size was increased to 100 participants to allow for the inclusion of all types of extraction [13].

### 2.2. Ethics Considerations

**Figure 1:**
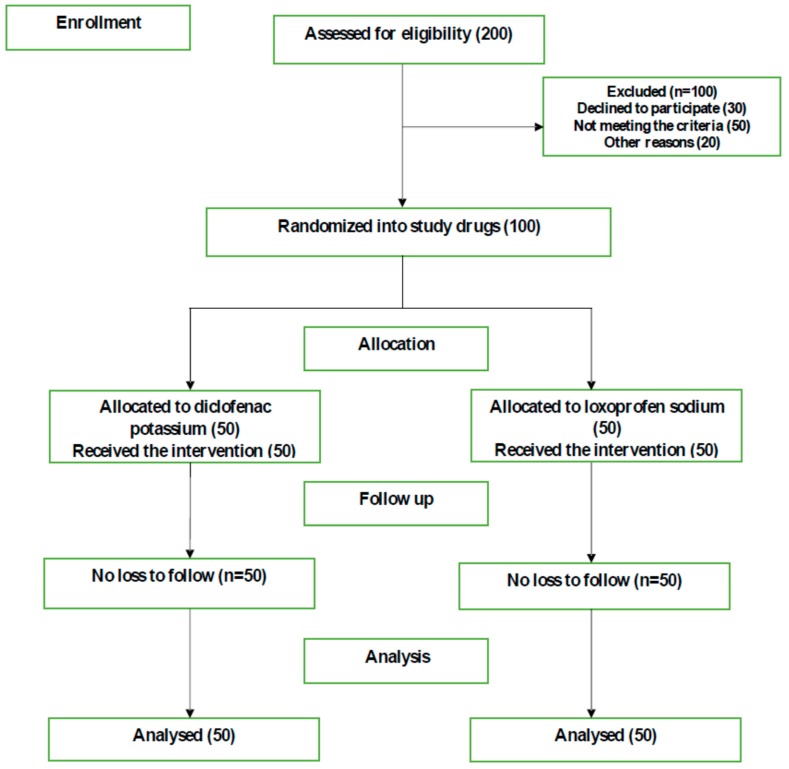
CONSORT flow chart diagram.

The study was approved by the Taibah University College of Dentistry Research Ethics Committee (TUCDREC/20180102/Alaufi, Date: 1 February 2018). The study was conducted in accordance with the principles of the World Medical Association of Helsinki; written consent was obtained from the participants after they were informed of the study’s objectives, methods and possible risks. Participation in this study was voluntary and patients had the right to withdraw at any time, without changes to the quality of their treatment. Confidentiality was assured, as every questionnaire was anonymous and coded (i.e., participant information was not identified). The study was registered at the International Standard Randomized Controlled Trials Registry (http://www.isrctn.com/ISRCTN62004181) and adhered to CONSORT guidelines (Figure 1).

### 2.3. Patients’ Recruitment and Inclusion and Exclusion Criteria

All patients, regardless of gender (Figure 1), who attended and were referred to surgical dental clinics for tooth extraction (simple or surgical extraction) every Monday and Wednesday over the aforementioned period were invited to participate in the study. Participants had to meet the following selection criteria: ages 18–70 years old, literate (speaking, reading and writing Arabic or English), healthy or with controlled systemic disease as recommended by the American Society of Anesthesiologists and no risk from the administration of local anesthesia (LA) with adrenaline (i.e., hyperthyroidism) [14]. Patients were excluded if they could not give informed consent (e.g., mental disorder), had teeth with reversible pulpitis and/or had a history of taking anticoagulants, active peptic ulcers and/or asthma attacks.

### 2.4. Randomization and Allocation into the Intervention 

Eligible patients were randomized into the study’s two intervention groups (diclofenac potassium and loxoprofen sodium) by means of drawing lots. The two groups were coded as A and B. The codes of the drugs were kept by an independent monitor and were not revealed until after data analysis. The researcher, clinicians and patients were blinded to the codes of the drugs A and B during the course of the study. When the participant opened the envelope, he/she found instructions on how to take the medicine according to the manufacturer’s specifications.

### 2.5. Procedure of Extraction

In oral surgery, administration of LA is considered a common procedure before tooth extraction [15]. For the procedure of exodontia, LA (1.8 mL scandicaine 2% with epinephrine 1:100,000) was administered to the patient seated in a dental chair. For upper teeth, patients were given buccal infiltrations, and for lower teeth, either buccal infiltrations or inferior alveolar nerve block (IANB). Elevators and dental forceps were used to complete the extraction. The researcher was just an observer. Pain was assessed 6 h post-operatively and then every 12 h for 3 days. As discussed, two randomly distributed analgesics (loxoprofen sodium and diclofenac potassium) were used in this study. Loxoprofen sodium (Roxonin 60 mg) is a tablet used for post-extraction pain and inflammation [12], and diclofenac potassium (Rapidus 50 mg) is used to relieve pain and swelling (inflammation) [6].

### 2.6. Assessment of Post-Operative Dental Pain 

The pain assessment was carried out 6 hours post-dental extraction and every 12 h thereafter on the same day of extraction and the following two days, because the highest pain point is between 6 to 8 h after extraction and intensity of pain has been shown to reach its maximum in the first 24 h [5,6]. The pain assessment was recorded by the patient via a questionnaire (data collecting sheet) using the Visual Analogue Scale (VAS). The VAS provides a simple technique for measuring pain intensity. It is valid and reliable in a range of clinical and research applications. The pain VAS is a continuous scale comprising a horizontal (HVAS) or vertical (VVAS) line, usually 10 centimeters in length, with two endpoints: “no pain” and “extremely painful” [16,17]. However, in this study, the Verbal Descriptor Scale (VDS) was utilized to facilitate the report of pain among patients who were anticipated to have difficulty conceptualizing the VAS, as well as to ensure participants’ comprehension, understanding and collaboration [18,19]. Notably, the World Health Organization (WHO) has used VDS as a replacement of VAS in studies focusing on analgesic scale in cancer patients [20]. The VDS is composed of four categories: 0 = no pain, 1–4 = mild pain, 5–7 = moderate pain and 8–10 = severe pain.

### 2.7. Data Analysis

The statistical package for social sciences version 16 (SPSS Inc., Chicago, IL, USA) was used for data analysis. The categorical data (e.g., gender) was presented as frequency with percentages (F [%]). Bivariate analysis chi-squared tests or Fisher’s exact test were run as appropriate to identify any statistically significant association between type of drug and post-operative severity of pain as well as to identify differences in pain severity within each drug group (male, female). The “no pain” and “mild pain” were collapsed into simply “no pain,” and “moderate pain‘‘ and ‚‘severe pain” into “pain” due to the small sample size and to ease interpretation of the results. The difference was considered significant at *p* < 0.05. To allow for the discussion of the post-operative pain, the drug codes (A = loxoprofen sodium, B = diclofenac potassium) were released at the completion of the data analysis by the independent personnel.

## 3. Results

### 3.1. Sample Characteristics

Of the 100 recruited patients, 54 (54%) were males and 46 (46%) females. The age groups of participating patients were as follows: 18–30 years old (26%), 31–40 years old (24%), 41–50 years old (29%) and 50–70 years old (21%). As shown in Table 1, there was non- significant difference (*p* = 0.688) for gender randomized in both drugs nor for age (*p* = 0.449). Of the patients in the study sample, 80% (*n* = 80) used the drugs after simple extraction (anterior and posterior teeth) and only 20% (20) patients used these drugs for other types of extractions of posterior teeth (surgical, 12% [12] and surgical with flaps, 8% [8]). Comparable percentages (*p* = 0.317) of the latter types of extractions were randomized into both drug groups.

### 3.2. Descriptive and Bivariate Comparisons of Loxoprofen and Diclofenac Post-Operative Pain Relief 

Post-operative pain was reported by the patients on the day of the extraction and the following two days. Comparable percentages of “no pain” perception 6 hours after extraction were reported by both drug groups (*p* =0.398). The percentage of “no pain” perception was increasingly reported over time for both drugs. As shown in Table 2, there were almost non-significant differences (*p* > 0.05) in post-operative pain perceptions among the loxoprofen sodium and diclofenac potassium groups over the allocated time intervals, except on day two (after 36 h). A statistically significantly number of patients receiving diclofenac potassium reported “no pain” compared to patients receiving loxoprofen sodium drug (86% vs. 66%, respectively, *p* = 0.019).

### 3.3. Pain Perception within Each Drug Group (Male, Female) 

For the comparison of the post-operative pain perception between genders within each drug group, there were non-significant differences (*p* > 0.05) between males and females for all time intervals (6 h after extraction, 24 h after extraction and onwards).

## 4. Discussion

It is worth noting that “pain is a complex unpleasant experience with complicated interactions of physiological and psychological components” [21]. This study compared two NSAIDs (loxoprofen and diclofenac) based on clinical efficacy rather than in vivo measurement of COX enzyme inhibition and prostaglandin concentration in the tissue. Despite the subjectivity of pain sensation, we are describing the first study in Saudi Arabia that has assessed the efficacy of loxoprofen sodium and diclofenac potassium drugs in post-operative dental pain relief among male and female patients with simple and surgical tooth extraction. Generally speaking, the two randomly distributed analgesics (loxoprofen sodium, diclofenac potassium) used in this study both showed a significant reduction in post-operative pain relief.

However, with respect to the proposed hypothesis of post-operative pain relief, diclofenac potassium demonstrated better post-operative pain relief compared to loxoprofen sodium, particularly on day two (36 h post-operation). Our findings align with previous studies that have discussed the differences between some NSAIDs. One such study compared ibuprofen, paracetamol and diclofenac potassium after deep cavity preparation and third molar extraction, in which it was concluded that diclofenac potassium has better pain control over post-operative pain [6]. In addition, a double-blind, randomized controlled trial completed in 2019 comparing the effectiveness of celecoxib and tramadol after third molar extraction concluded that celecoxib, which is a selective COX-2 inhibitor NSAID, was more efficacious and better tolerated than tramadol, which is considered a weak opioid [22]. This is also in line with studies demonstrating that post-operative dental pain is associated with prostaglandin peripheral release rather than opioid central action [7]. A meta-analysis of 76 studies reported that diclofenac has greater analgesic effectiveness compared to eight other NSAIDs [21]. On the other hand, a study comparing the effectiveness of three intramuscular drugs regarding pain relief—diclofenac potassium, naproxen sodium and etodolac—showed no difference regarding pain relief among the drugs [23]. The discrepancy in results reflects the degree of COX inhibition by NSAIDs, variable underlying pain mechanisms, imprecision of pain scales and cultural norms of pain expression. 

Furthermore, comparing our results with a randomized, triple-blind clinical study comparing the efficacy of an NSAID drug and a combination of NSAID plus tramadol/acetaminophen showed that both groups had good pain control after lower third molar extraction and that the difference is not clinically relevant [24]. Additionally, though the literature reported that females have lower thresholds of pain than males [11], the two drugs were comparable with respect to their effects on males and females participating within the same drug groups. 

The strengths of the study were as follows: First, the drugs used were triple blinded (surgeons, patients and researchers), which likely reduced assessment bias and increased the accuracy and objectivity of clinical outcome findings. Second, both genders and types of extractions (simple or surgical) were included. Third, we adopted a patient-centered health outcomes assessment in the form of patient-reported post-operative pain. As for the study limitations, the sample size was considered small (i.e., categories of post-operative pain were collapsed to aid data analysis and results interpretation). In addition, the use of the Visual Descriptive Scale for pain assessment instead of the Visual Analogue Scale may have obscured a thorough assessment of pain trends; however, we anticipated that patients attending the service would be less likely to conceptualized the numerical and linear scale. Finally, confounding factors such as smoking, socioeconomic status and patient weight and their role in pain perception were not investigated within the context of the study.

## 5. Conclusions

Among this study sample, the two randomly distributed analgesics (loxoprofen sodium, diclofenac potassium) were both shown to significantly reduce post-operative pain irrespective of gender or type of dental extraction. Notably, diclofenac potassium demonstrated slightly better post-operative pain relief than loxoprofen sodium. 

## Figures and Tables

**Table 1:** Characteristics of the partcipants (*n* = 100).

Variable	Total F (%)	Loxoprofen Sodium F (%)	Diclofenac Potassium F (%)	*p-*Value ^a^
**Gender**				
Male	54 (54)	26 (52)	28 (56)	0.668
Female	46 (46)	24 (48)	22 (44)	
**Age**				
18–30 years old	26 (26)	14 (28)	12 (24)	
31–40 years old	24 (24)	11 (22)	13 (26)	0.499
41–50 years old	29 (29)	17 (34)	12 (24)	
51+ years old	21 (21)	8 (16)	13 (26)	
**Type of Extraction**				
Simple	80 (80)	38 (76)	42 (84)	0.317
Surgical ^b^	20 (20)	12 (24)	8 (16)	

^a^ Chi-square test; ^b^ Surgical or surgical with flab.

**Table 2:** Comparisons of loxoprofen sodium and diclofenac potassium post-operative pain perception (*n* = 100).

Variable	No Pain ^†^	Pain ^∞^	*p-*Value
**6 h after extraction**			
Loxoprofen Sodium	31 (62.0)	19 (38.0)	0.398 ^a^
Diclofenac Potassium	35 (70.0)	15 (30.0)	
**24 h after extraction**			
Loxoprofen Sodium	36 (72.0)	14 (28.0)	0.148 ^a^
Diclofenac Potassium	42 (84.0)	8 (16.0)	
**36 h after extraction**			
Loxoprofen Sodium	33 (66.0)	17 (34.0)	0.019 ^a^
Diclofenac Potassium	43 (86.0)	7 (14.0)	
**48 h after extraction**			
Loxoprofen Sodium	41(82.0)	9 (18.0)	0.065 ^a^
Diclofenac Potassium	47 (94.0)	3 (6.0)	
**60 h after extraction**			
Loxoprofen Sodium	43 (86.0)	7 (14.0)	0.182 ^a^
Diclofenac Potassium	47 (94.0)	3 (6.0)	
**72 h after extraction**			
Loxoprofen Sodium	44 (88.0)	6 (12.0)	0.112 ^b^
Diclofenac potassium	49 (98.0)	1 (2.0)	

^†^ No pain = “no pain” and “mild pain”; ^∞^ Pain = “moderate pain ‘‘and “severe pain”; ^a^ Chi-square test; ^b^ Fisher’s exact test.
